# Molecular Biomarkers in Cutaneous Photodynamic Therapy: A Comprehensive Review

**DOI:** 10.3390/diagnostics14232724

**Published:** 2024-12-03

**Authors:** Jorge Naharro-Rodriguez, Stefano Bacci, Montserrat Fernandez-Guarino

**Affiliations:** 1 Programa de Doctorado en Ciencias de la Salud, Universidad de Alcalá de Henares, 28801 Madrid, Spain; 2 Dermatology Department, Ramon y Cajal University Hospital, 28034 Madrid, Spain; drafernandezguarino@gmail.com; 3 Research Unit of Histology and Embriology, Department of Biology, University of Florence, 50139 Florence, Italy; stefano.bacci@unifi.it

**Keywords:** photodynamic therapy, dermatology, biomarkers, histology

## Abstract

Background/Objectives: Photodynamic therapy (PDT) is widely utilized in dermatology for the treatment of various skin conditions. Despite its effectiveness, the exact biomolecular changes underlying therapeutic outcomes remain only partially understood. This review, through a transversal approach, aims to provide an in-depth exploration of molecular biomarkers involved in PDT, evaluate its underlying mechanisms, and examine how these insights can contribute to enhanced treatment protocols and personalized therapy approaches. Methods: A narrative review of the literature was conducted, targeting peer-reviewed articles and clinical trials that focus on PDT and its molecular biomarker effects on dermatological conditions. The databases searched included PubMed, Scopus, and Web of Science, and the inclusion criteria encompassed original research articles, systematic reviews, and meta-analyses in English. Results: PDT effectively reduces the expression of critical biomarkers such as p53, Cyclin D1, and Ki-67 in AK and other cancerous lesions, leading to reduced cell proliferation and increased apoptosis. Additionally, PDT promotes extracellular matrix remodeling and stimulates collagen production, which has a rejuvenating effect on the skin and a promising role in the treatment of chronic wounds. Conclusions: PDT represents a powerful and versatile treatment option for various dermatological conditions due to its ability to target cellular pathways involved in proliferation and apoptosis. Further research into optimizing treatment parameters and combining PDT with other targeted therapies may enhance patient outcomes, reduce resistance, and pave the way for more individualized therapeutic approaches in dermatology.

## 1. Introduction

Photodynamic therapy (PDT) is a well-known technique widely used by dermatologists for the treatment of non-melanoma skin cancer and precancerous conditions. The main advantage of PDT is the treatment of tumoral and precancerous skin lesions while avoiding surgical excision. This approach is indicated for non-high-risk tumoral lesions with an excellent cosmetic result [1,2]. Regarding precancerous skin lesions, such as actinic keratosis (AK), PDT allows the treatment of extended field cancerization areas with approximate cure rates of 80%, making it one of the most effective treatments for this condition [3]. There is some evidence of PDT’s capacity to prevent non-melanoma skin cancer, but AK progression remains controversial [4].

PDT in dermatology consists of the topical application of a photosensitizer (FS) under occlusion and illumination with an optimal light source. Irradiation after the absorption of the FS allows the destruction of the targeted cells [5]. A wide variety of FS and light sources has been proven effective in PDT [6]. In general, in dermatology, topical FS are preferred, as they are simpler to apply and produce fewer secondary effects compared to systemic FS.

The mechanism of cell destruction in PDT is based on the production of radical oxygen singlet (ROS) species using the mitochondrial bias; however, some indirect tissular mechanisms in PDT have been described [6]. It has been postulated that different mechanisms of action could be enhanced by selecting different intensities, doses, or light parameters in PDT, and thus improve results in patients [7].

Histological studies of lesions treated with PDT showed cell destruction through both apoptosis and necrosis [8]. These are traditionally considered direct effects of PDT [9], but other indirect mechanisms have been described. PDT has been proven to affect vessel regulation and angiogenesis [10], promote extracellular matrix regeneration [11], and develop immunological local effects [12]. Understanding variations in histological markers following PDT helps in comprehending its molecular mechanisms, thereby improving the technique and clinical outcomes, as these depend on the parameters of the light used and the FS [13].

The aim of this review is to comprehensively assess the role of molecular biomarkers in the efficacy of photodynamic therapy (PDT) in various dermatological conditions in a transversal way, including actinic keratosis, non-melanoma skin cancer, skin rejuvenation, and wound healing. For each application, existing evidence on their underlying molecular alterations will first be reviewed from a diagnostic perspective, followed by a discussion of the therapeutic effects of photodynamic therapy at the molecular level in these conditions. This review seeks to provide a deeper understanding of the mechanisms underlying PDT and its clinical impact. The review was conducted using a narrative approach to identify relevant studies on molecular biomarkers in PDT. Inclusion criteria included peer-reviewed articles and clinical trials focusing on PDT and its effects on molecular biomarkers in dermatological conditions. Studies were excluded if they did not provide specific data on biomarker modulation or if they were case reports, editorials, or conference abstracts. The search terms used included “photodynamic therapy”, “molecular biomarkers”, “immunochemistry”, “dermatology”, “actinic keratosis”, “basal cell carcinoma”, and “skin rejuvenation”. Only articles written in English were considered for inclusion. The databases searched included PubMed, Scopus, and Web of Science. The review focused on original research articles, systematic reviews, and meta-analyses. Sources were identified through database searches as well as by screening reference lists of relevant articles. Through this strategy, we aim to identify patterns in biomarker modulation that could help optimize PDT protocols, improve patient outcomes, address challenges such as treatment resistance, and assist in the development of an individualized medical approach.

## 2. Actinic Keratosis and Field Cancerization

Actinic keratosis (AK) is a common premalignant lesion that can develop into squamous cell carcinoma (SCC) if left untreated. It frequently occurs in sun-exposed skin, particularly in older individuals, and is characterized by dysplastic changes within the epidermis [14]. Photodynamic therapy (PDT) is a highly effective treatment modality for actinic keratosis (AK) and its associated cancerization field [15,16,17,18,19,20,21].

### 2.1. Molecular Biomarkers

The p53 tumor suppressor gene is a crucial molecular biomarker involved in AK and SCC development. Known as the “guardian of the genome”, p53 plays a pivotal role in regulating the cell cycle, DNA repair, and apoptosis in response to genotoxic stress such as UV radiation, which is a primary cause of skin cancer. When the skin is exposed to UVB radiation, pyrimidine dimers form in the DNA, and, if not repaired, these dimers lead to mutations [22]. One of the earliest events in UV-induced skin carcinogenesis is the mutation of the p53 gene, which prevents the proper function of this tumor suppressor, allowing damaged cells to survive and proliferate.

In healthy skin, p53 activation leads to either the repair of DNA damage or the initiation of apoptosis if the damage is too severe. In contrast, in AK lesions, p53 is frequently mutated, which diminishes its ability to regulate cell death and allows cells with DNA damage to survive. Mutations in p53 are not exclusive to AK lesions but also occur in sun-exposed skin that appears clinically normal. In this subclinical field cancerization, the presence of mutated p53 is a marker of early, invisible damage. It has been observed that more than 50% of AKs harbor mutated p53, and this number rises significantly in SCC, where up to 90% of cases exhibit p53 mutations [14].

Cyclin D1 is another critical molecular marker implicated in the development of AK and SCC. Cyclin D1 is a regulatory protein that controls the transition from the G1 phase to the S phase of the cell cycle, facilitating DNA replication and cell division. Overexpression of cyclin D1 accelerates cell cycle progression, shortening the G1 phase and promoting uncontrolled proliferation, which is a hallmark of cancerous growth. This dysregulation of the cell cycle is a key event in the progression from pre-cancerous lesions like AK to invasive SCC. Cyclin D1 is overexpressed in approximately 50% of AK lesions, indicating its significant role in early skin carcinogenesis [23]. Cyclin D1 overexpression is not limited to pre-cancerous conditions; it is also present in a wide range of malignant conditions, including breast, esophageal, and liver cancers, where it is often used as a prognostic marker [24].

Ki-67 is a well-established marker of cellular proliferation, expressed in actively dividing cells but absent in quiescent (non-dividing) cells. In AK and SCC, high levels of Ki-67 expression are indicative of increased cell turnover and are associated with the rapid proliferation of keratinocytes, which drives lesion development. Ki-67 expression levels can assess the aggressiveness of AK lesions, with higher levels correlating with an increased risk of progression to SCC [25].

Ki-67 is also found in the cancerization field, suggesting that even skin that appears clinically normal may harbor areas of heightened cellular activity that are at risk of progressing to overt malignancy.

The Fas/Fas ligand (Fas/FasL) system is a critical regulator of apoptosis. In the extrinsic apoptotic pathway, the binding of FasL to its receptor Fas (CD95) on the cell surface triggers the activation of caspase-8, which then activates downstream caspases, such as caspase-3, leading to cell death. This pathway is particularly important in preventing the survival of damaged cells that might otherwise accumulate mutations and progress to cancer [14].

In the context of AK and the cancerization field, dysregulation of the Fas/FasL pathway can impair the ability of keratinocytes to undergo apoptosis in response to DNA damage, allowing these cells to survive and contribute to the development of SCC. Mutations in key components of the apoptotic machinery, such as Fas or caspases, can result in a reduced capacity for cell death and increased resistance to therapies that rely on apoptosis induction, such as PDT [25].

Survivin, a member of the inhibitor of apoptosis (IAP) family, is implicated in both the regulation of apoptosis and cell division. Survivin inhibits caspase-9 and blocks the mitochondrial pathway of apoptosis, thereby promoting the survival of damaged cells. Its overexpression in AK and SCC lesions is associated with resistance to apoptosis and increased tumor cell survival. Moreover, survivin is linked to treatment resistance, particularly in therapies such as radiotherapy. In the context of PDT, survivin presents a potential obstacle to achieving complete tumor regression, as its anti-apoptotic effects may reduce the efficacy of the therapy [26,27].

### 2.2. Biomolecular Impact of PDT

Photodynamic therapy has a profound impact on p53 expression in AK and the surrounding cancerization field. Studies have demonstrated that PDT reduces the accumulation of mutated p53 protein in keratinocytes, which correlates with a reversal of the carcinogenic process. However, the effectiveness of PDT in completely eliminating p53-mutant cells can vary depending on the extent of the damage and the depth of the lesions. In some cases, residual p53-positive cells may persist after a single treatment. Additionally, the ability of PDT to reduce p53 expression may also be influenced by the type of photosensitizer used, the dose of light administered, and the specific characteristics of the lesion. A study by Bagazgoitia et al. demonstrated that while PDT reduces p53 expression, residual expression may persist in some lesions, indicating the need for multiple treatments to achieve complete resolution [28].

PDT has been shown to significantly reduce the expression of both cyclin D1 and Ki-67, key markers of cellular proliferation in AK lesions. By halting the cell cycle and promoting apoptosis, PDT reduces the proliferative activity of keratinocytes, leading to the regression of pre-cancerous lesions. Studies have shown that PDT leads to a marked decrease in Ki-67 expression, reflecting a reduction in the number of actively dividing cells in the treated area [14,23].

While cyclin D1 levels also decrease following PDT, it has been observed that this reduction may not be as complete as that of Ki-67. In some cases, cyclin D1 expression persists after treatment, particularly in more advanced or thicker AK lesions. This suggests that while PDT is effective in reducing cellular proliferation, additional treatments or combination therapies may be needed to fully suppress cyclin D1 activity and ensure complete remission [23].

PDT has been shown to directly modulate the Fas/FasL pathway, enhancing the expression of Fas and FasL on the surface of tumor cells and inducing apoptosis. The activation of this pathway is an early event following PDT, occurring within hours of light activation. Studies have demonstrated that PDT-sensitized tumor cells exhibit increased levels of Fas and FasL, leading to the activation of caspase-8 and downstream effector caspases such as caspase-3, which are crucial for the execution of apoptosis [29].

By promoting apoptosis through the Fas/FasL pathway, PDT effectively eliminates damaged keratinocytes in both the visible AK lesions and the surrounding cancerization field. The upregulation of Fas signaling post-PDT precedes mitochondrial events such as cytochrome c release, which further amplifies the apoptotic signal and ensures the removal of damaged cells [29].

One of the challenges in PDT treatment is overcoming the resistance conferred by survivin, which inhibits apoptosis and promotes cell survival. Survivin expression is upregulated following PDT, particularly in the surviving tumor cells, which can limit the overall effectiveness of the therapy. However, studies have shown that combining PDT with survivin inhibitors, such as 17-AAG, can significantly enhance the therapeutic effects by promoting the degradation of survivin and other anti-apoptotic proteins [30].

Inhibition of survivin following PDT leads to increased apoptosis and reduced tumor cell survival, suggesting that targeting survivin may be a valuable strategy for improving PDT outcomes. This combination approach has shown promise in preclinical studies, where survivin inhibition enhances caspase activation, increases the cleavage of PARP (a marker of apoptosis), and improves overall cytotoxicity in treated cells [27].

In addition to its molecular effects, PDT induces significant histopathological changes in AK lesions and the surrounding cancerization field. Following PDT, treated skin exhibits a reduction in dysplasia, with a marked decrease in the number of atypical keratinocytes. Necrosis of the epidermal layers is commonly observed, accompanied by apoptosis and the removal of damaged cells [14].

Campione et al. demonstrated that PDT reduces the expression of matrix metalloproteinases (MMP-1 and MMP-2) in AK lesions. MMPs are known to degrade the extracellular matrix (ECM) and promote tumor progression. Their reduction after PDT suggests a molecular shift towards the stabilization of the ECM, preventing further degradation and reducing the potential for AK to progress to invasive squamous cell carcinoma (SCC). The findings indicated a significant improvement in skin architecture, as observed through histopathological and immunohistochemical analyses, further supporting the role of PDT in restoring tissue integrity [31]. Georgescu et al. assessed proangiogenic markers (MMP-2, MMP-9, VEGF, and FGF-2), antioxidant status, and hypoxia indicators (HIF-1 alpha) in AK and SCC. PDT significantly downregulated these markers in AK lesions, reducing angiogenesis and oxidative stress. Since angiogenesis is a key factor in tumor progression, its reduction after PDT implies a decreased risk of AK progression to SCC [32].

In the days following treatment, regenerative changes become apparent, with the proliferation of healthy keratinocytes and the restoration of normal skin architecture. Over time, these histopathological improvements contribute to the overall clinical efficacy of PDT, as the treated areas become less likely to progress to invasive SCC. However, as with molecular changes, complete histological resolution may require multiple treatment sessions, particularly in areas with extensive field cancerization [22].

A recently published study performed a transcriptomic analysis of AK lesions, identifying two molecular subclasses: AKs with profiles similar to SCCs (“lesional AKs”) and those with profiles similar to normal skin (“non-lesional AKs”). PDT likely targets these “lesional AKs” more effectively, reducing the expression of genes associated with inflammation and ECM degradation. The identification of these molecular subclasses emphasizes the heterogeneous nature of AKs and the need for personalized treatment approaches [33].

Table 1 summarizes the biomarkers discussed in this section and their modifications following PDT.

## 3. Skin Remodeling and Rejuvenation

### 3.1. Molecular Biomarkers

During skin aging, collagen type I is naturally degraded by matrix metalloproteinases (MMPs), a process accelerated by sun exposure. This destruction is part of the so-called photoaging and is more intensive in fair skin. Collagen type I is the most important structural protein of the skin matrix in the dermis, which is under continuous renovation promoted by MMPs, slowly fragmented and substituted by new cross-linked collagen. Skin senescence impairs collagen type I renewal, causing a defective dermis due to the accumulation of irreparable collagen [34]. MMPs are produced by fibroblasts, which, during senescence, suffer progressive dysfunction supported by two theories: on the one hand the reduction of their proliferative capacity and MMP production, and on the other hand, the aerobic metabolism induced by reactive oxygen species (ROS) [35]. There are four types of MMPs, from 1 to 4, with MMP-1 being the most implicated in collagen substitution.

### 3.2. Biomolecular Impact of PDT

PDT exhibited the ability to induce skin remodeling and thus photo-rejuvenation in in vitro studies. Karrer et al. were among the first researchers to publish this observation. In an in vitro experiment, keratinocytes were treated with PDT, showing an increase in IL-1-alpha, TNF-alpha, and IL-3, but not in the levels of MMPs. Nevertheless, when fibroblasts were exposed to keratinocytes treated with PDT, a significant increase in MMP-1 and MMP-3 was detected [11]. On the other hand, when fibroblasts were treated with PDT, a significant induction of MMP-1 and MMP-3 was detected after 6–72 h. For the authors, these findings demonstrated that PDT induction of MMPs is both direct through fibroblasts and indirect via keratinocytes. These in vitro data were confirmed in a subsequent investigation conducted by Kim et al., which also found indirect effects of PDT on fibroblasts caused by the cytokines released by keratinocytes, including IL-1-alpha, TNF-alpha, and IL-6 [36].

Histological changes in the dermis after PDT, when a complete tumoral response is induced, are detected 7 days after treatment, when the dermis shows an increase in fibroblasts as well as new collagen formation mixed with an inflammatory lymphocytic infiltrate. After 4 to 8 weeks the dermis shows an increase in collagen with a complete restoration of the epidermis, flattening the ridges caused by fibrosis [37].

Zhang et al. discussed the positive effects of low-dose photodynamic therapy using aminolevulinic acid (ALA) and its methyl ester (MAL). Low-dose PDT was found to promote fibroblast proliferation, inhibit DNA damage, counteract oxidative stress, and modulate inflammatory processes—all of which contribute to skin rejuvenation. Low concentrations of reactive oxygen species (ROS) generated during low-dose PDT act as a signaling molecule, promoting cellular activities beneficial for skin rejuvenation, such as collagen production and the remodeling of aged skin [38].

Papayan et al. utilized skin autofluorescence spectroscopy (SAF) to assess the molecular changes in the skin during PDT for rejuvenation. The authors reported that PDT resulted in a significant reduction in advanced glycation end products, lipofuscin-like lipopigments, and porphyrins. The reduction of AGEs, which are linked to aging and photoaging, was notable for both types of bonds—with collagen and elastin. This implies a rejuvenating effect at the structural protein level, potentially improving skin elasticity and reducing wrinkles. The use of chlorin e6 as a photosensitizer and low-intensity light led to changes in the skin’s biochemical profile, contributing to a younger appearance [39].

Another study evaluated the effect of combining PDT with intense pulsed light (IPL) for photodamaged skin. The study demonstrated superior outcomes in skin rejuvenation when PDT using 10% ALA gel was combined with IPL, compared to PDT alone. Molecularly, the treatment promoted photorejuvenation by reducing elastotic material and enhancing neocollagenesis, which led to the improvement of skin texture, tone, and elasticity. Additionally, inhibition of melanogenesis through modulation of tyrosinase activity and related cytokine signaling helped reduce dyspigmentation, providing a more uniform skin tone [40].

Yan et al. used single-cell RNA sequencing to reveal the impact of ALA-PDT on the cutaneous immune microenvironment. The study found that ALA-PDT increased the proportion of active immune cells, improved cell-cell communication, and restored functions such as antigen presentation and migration of dendritic cells—all of which are crucial for maintaining healthy, youthful skin. This immunomodulatory effect of PDT may play a role in sustaining skin health over extended periods [41].

In vitro studies have shown that PDT may promote the initial repairing process to heal photodamaged skin, stimulating collagen type I and III synthesis and inducing the secretion of MMPs to remove photodamaged collagen fibers [11,42]. These studies were conducted using low doses of PDT and thus were supposed to occur surrounding the treated lesions [36]. Afterwards, the levels of collagen increase in the dermis, along with the expression of TGF-beta, which increases collagen proliferation. At the end of the repair process photodamaged collagen fibers are removed, replaced with new collagen and the dermis becomes thinner [37].

These findings have been correlated with histological and immunohistochemical examination with biopsies of human skin before and after PDT [43]. In healthy skin biopsies, 3 weeks after PDT, higher expression of MMP-1, MMP-3, and IGF-beta was detected, indicating matrix remodeling. Moreover, after 9 months, treated areas exhibit ordered and significantly higher quantities of collagen I, collagen III, and elastin, along with a decrease in MMP expression [44]. Almeida et al. studied biopsies after PDT, observing that an increased expression of MMP-9 was detected 3 months after treatment, as well as an increase in collagen type I [45] (summarized in Table 2)

## 4. Wound Healing

The process of wound healing is categorized into four sequential phases: hemostasis, inflammation, proliferation, and remodeling/maturation [46,47].

During hemostasis, endothelial cells secrete von Willebrand factor, inducing platelet attachment and fibrin clot formation. This process causes smooth muscle to contract due to increased calcium ions, causing blood vessels to narrow and reduce blood flow. This leads to the generation of vasoactive metabolites, which widen and relax arterial blood vessels, lasting several minutes [46,47,48]. For other details, see Table 3.

During the inflammatory phase, mast cells (MCs) release histamine or serotonin, causing vasodilation and diapedesis, which involves the migration of neutrophil granulocytes and monocytes. This enhances phagocytosis, eliminating infections or damaged cells. Leukocytes release cytokines and growth factors, and keratinocytes contribute by generating inflammatory cytokines. Other molecules, such as cytokines, matrix proteins, and enzymes, also play a role in the inflammatory phase. Chemokines are essential for attracting neutrophils and lymphocytes to coordinate wound healing [48,49]. For other details, see Table 3.

Fibroblasts during the proliferative phase create granulation tissue, regulate keratinocyte migration and proliferation, and participate in angiogenesis. Macrophages secrete growth factors engaged in this phenomenon. For other details, see Table 3.

The maturation phase of healing involves the repair of collagen and the contraction of the wound, facilitated by myofibroblasts. The remodeling phase is regulated by growth factors like transforming growth factor (TGF)-beta signaling and Notch pathways, which control transitions between mesenchymal-mesenchymal and endothelial-mesenchymal phenotypes. Beta-2AR is a crucial molecule in facilitating the epithelial mesenchymal transition (EMT) process, and these changes occur via TGF beta-signaling or Notch pathways, suppressing cadherin expression in endothelial cells [50,51,52]. For other details, see Table 3.

The formation of scars is a process that requires the reorganization of granulation tissue. Matrix metalloproteinases (MMPs) and their inhibitors, such as tissue inhibitors of metalloproteinases (TIMPs), play an important role in this phenomenon. A decrease in the synthesis of extracellular matrix (ECM) and changes in its contents, such as the substitution of type III collagen for type I collagen, are the results of this process. In granulation tissue, elastin, which had been lacking in the past, is now present [53].

### 4.1. Molecular Biomarkers

The process of wound healing is primarily controlled by the secretion of cytokines and growth factors. Any deviation from it could lead to the development of a chronic wound. The pro-inflammatory cytokines, more specifically IL-1beta, TNF-alpha, and IL-6, play a significant role in the process of recruiting inflammatory cells to the location of the lesion. The inflammatory cells that are present at the site of the damage secrete a variety of growth factors, such as TGF-beta and Platelet-Derived Growth Factor (PDGF), which attract the fibroblasts that are proliferating to that particular region. In order to encourage the expansion of epithelial cells, macrophages and active fibroblasts secrete a number of growth factors, including fibroblast growth factor 2 (bFGF), keratinocyte growth factor (KGF), FGF-7, epithelial growth factor (EGF), hepatocyte growth factor (HGF), TGF-alpha, and insulin-like growth factor (IGF) 1. Macrophages, fibroblasts, and keratinocytes are responsible for the production of Vascular Endothelial Growth Factor (VEGF) and PDGF, which are cytokines that encourage endothelial cells to initiate the process of angiogenesis [54,55].

Other factors that contribute to this process include transcription (specifically the E2F family) and signaling (Wnt/beta-catenin), as well as Signal Transducer and Activator of Transcription (STAT) 3, homeobox genes, hormone receptors (androgens, estrogens, and glucocorticoids), Peroxisome Proliferator-Activated Receptors (PPARs), Activator Protein 1 (AP-1), c-Myc, ETS-Related Gene (Erg) 1, proteases (including MMPs), cytoskeleton proteins, and enzymes involved in regulating the cellular redox balance. All of the components that have been stated are interconnected, which means that they are not independent of one another [54,55].

Both invertebrates and vertebrates display unique diffusible signals that are independent of transcription during the process of wound healing. Undoubtedly, hydrogen peroxide (H_2_O_2_) and adenosine (for additional autocrine production of ATP) have essential functions.

Protein Kinase C (PKC), Ca^2+^/Calmodulin-Dependent Protein Kinase (CaMK), and ROS generally alter genetic transcription due to the rapid increase in intracellular Ca^2+^ concentration caused by the lesion. This process is involved in various cellular functions such as cell communication, migration, adhesion, inflammatory responses, angiogenesis, and re-epithelialization. Additionally, it is important to take into account that tissue damage triggers the initiation of Ca^2+^ waves, which in turn activate the RHO family GTPases, leading to an increase in actin polymerization and actomyosin contractility. This process is crucial for maintaining the structural integrity of the stroma. In addition, the release of Ca^2+^ stimulates many signaling pathways, including c-Jun N-Terminal Kinase (JNK) and Mitogen-Activated Protein Kinase (MAPK), which activate transcription factors and boost the expression of genes involved in the response to insults, such as those related to the cytoskeleton. Purinergic receptors play a crucial role in the wound healing process by modulating the release and activation of ATP. Epithelial cells neighboring the insult detect DNA damage using P2Y receptors. These receptors have the ability to relay signals within the cell, which involve activating intracellular calcium ions (Ca^2+^) and MMPs. This mechanism guarantees the liberation of particular growth factors (such as EGF) that have the ability to activate the numerous cascade mechanisms involved in the process of wound healing [54,55,56].

#### 4.1.1. Genetic Activation in Wound Healing

The functionality of a number of genes that encode for particular molecules (cytokines, chemokines, and growth factors) defines the properties of the various stages of wound healing and the overlap between them. The genes Tyrosinase (TYR), Tyrosinase-Related Protein 1 (TYRP1), and Dopachrome Tautomerase (DCT) are examples of hub genes that play a role in the formation of melanin. Eighty-five differentially expressed genes (DEGs) and one hundred sixty-four proteins that were downregulated were found during the inflammatory and proliferative phases. There are three hub genes that are involved in the P53 signaling pathway and the cell cycle. These genes are referred to as Cyclin B1 (CCNB1), Checkpoint Kinase 1 (CHEK1), and Cyclin Dependent Kinase 1 (CDK1). In the course of the remodeling phase, a total of 121 DEGs and 49 weakly expressed genes were discovered. There is a relationship between the hub genes for Collagen Alpha Chain 1 (COL4A1), Collagen Type 4 Alpha Chain 2 (COL4A2), and Collagen Type 6 Alpha Chain 1 (COL6A1) and the digestion and absorption of proteins, as well as the interaction with the extracellular matrix receptor [57]. Additionally, it is essential to take into consideration that, over the course of the last few decades, scientific study has concentrated on the influence that each cytokine has on particular parameters of WH in a wide range of experimental situations. In recent studies, it has been revealed that the key genes that are involved in the connections between the IL-17 signaling pathways and the different receptors include IL-1Beta, IL-6, CCL4, CXCL1, CXCL2, CXCL3, CXCL5, CXCL6, and CXCL10. It has also been proven that IL-6 and IL-1beta are essential for the stimulation of keratinocyte motility and the repair of the epidermis. Both of these processes are essential for the skin. Last but not least, it is essential to point out that recent study has demonstrated that the low production of CXCL1 and CXCL5, which are chemoattractants for neutrophils, decreases the amount of white blood cells in mice [54,55,56].

Genes that are pro-inflammatory and are expressed in the early stages of injury are responsible for the activation of molecules such as TNF-alpha, Interferon (IFN) gamma, and TGF-beta. The gene profile includes genes that encode chemicals such as VEGF, PDGF, Fibroblast Growth Factor (FGF)2, and MMP. These substances possess the capacity to stimulate the growth of fibroblasts and keratinocytes, as well as the formation of new blood vessels and the regeneration of epithelial tissue, as the process of wound healing develops. In order to enable the creation of collagen by fibroblasts and the removal of ECM during tissue resorption, the genes that encode TGF-beta1 and MMP expression are increased during the remodeling phase. It is possible for any change in gene expression to have an effect on the healing sequence. This shift can lead to the release of substances such as cytokines, chemokines, and growth factors, which can ultimately result in the development of chronic lesions [54,55,56].

Additionally, epigenetic mechanisms are involved in the process of wound healing, despite the fact that the molecular mechanism is not fully understood. To this day, a wide range of instances have been amassed about these mechanisms, which has made it possible to find a wide range of microRNAs.

Furthermore, these microRNAs have a role in the control of inflammatory reactions, the production of extracellular matrix (ECM), the cellular proliferation, and the communication between cells that takes place during the processes that are associated with wound healing [54,55,56].

As an example of post-translational mechanisms, the proteolytic lysis of fibronectin, which contributes to the promotion of cell proliferation and migration throughout the process of wound healing [54,55,56], serves as an illustration.

According to what has been seen, the process of wound healing is characterized by a regulated quality feature that differs from person to person. It has been demonstrated that certain mouse lines, such as MRL/MpJ-Faslpr (MRLF), are able to repair an ear-punched hole with a diameter of 2 mm during a period of thirty days. On the other hand, other mouse lines, such as C57BL/6 or SJLJ, demonstrate a healing rate of forty percent and twenty-five percent, respectively, within the same period of time [54,55,56].

#### 4.1.2. Dysfunction of the Cellular Mechanisms Associated with Wound Healing

##### Chronic Wounds

In the case that the stages of wound healing are not finished within six to eight weeks, the wound is considered chronic, and the treatment for it is quite expensive [57,58,59,60,61,62]. Biofilm formation commonly delays wound healing, contributing to the progression from acute to chronic wounds. This is true despite the fact that there are many different types of chronic wounds. An important property that distinguishes wound microbial communities from other types of communities is the presence of a wide range of bacterial species at the site of infection [57,58,59,60,61,62].

Alterations in MMP secretions, as opposed to acute lesions, are the primary mechanism by which chronic wounds (CWs) keep the inflammatory stage alive. To add insult to injury, the cellular infiltration is made up of a large number of cells that are responsible for the excessive inflammatory response that occurs in CWs. Neutrophils are seen in high numbers in CWs, and they are responsible for the release of significant amounts of metalloproteinases. Not only do these enzymes damage the connective tissue matrix and elastase, but they also inactivate key factors that are essential in the healing process of wounds. These factors include PDGF and TGF-beta. In spite of this, it is necessary to take into consideration the cellular interactions that occur between keratinocytes and immune cells that are present in the cellular infiltrate. Keratinocytes release a wide range of signaling molecules. However, it is still not clear how much these processes help contribute to CWs. Furthermore, since keratinocytes in CWs express genes that are associated with an incomplete proliferative activity, this gives an explanation for the enhanced proliferation of the epidermis near the borders of the ulcer. To add insult to injury, fibroblasts do not exhibit any migratory responses in response to TGF-beta stimulation. As a matter of fact, it has been observed that levels of TGF-betaR and the downstream components of the TGF-betaR signaling cascade have decreased [57,58,59,60,61,62]. As a last point of consideration, it is essential to keep in mind that neuroimmunomodulation has the potential to play a substantial role in the regulation of the cicatricial processes that occur in chronic wounds. Recently, cellular interactions between MC and neurons that contain mediators that are involved in processes associated with wound healing have been reported. The calcitonin gene-related peptide (CGRP), nerve growth factor (NGF), neurokinin A (NKA), neuropeptide Y (NPY), substance P (SP), protein gene product (PGP) 9.5, vasoactive intestinal peptide VIP [48], and nitric oxide (NO) are all examples of mediators. It is hypothesized that the cellular interaction between neurons and immune system cells may shed light on some phenomena that have been observed in the past. These phenomena include the excessive production of ECM by fibroblasts, the reaction of cellular infiltrates, and the elevated levels of TGF-beta [57,58,59,60,61,62].

### 4.2. Biomolecular Impact of PDT

The use of photodynamic therapy (PDT) to reduce all types of microorganisms that induce ROS and to prevent the development of resistance to conventional antibiotics has been investigated in the context of CWs. Additionally, the reduction in MMP activity and the regeneration of collagen must be taken into account through PDT-induced tissue regeneration. Nevertheless, the utilization of PDT as an assisted CW in clinical practice is not yet a common practice due to the scarcity of published studies and the necessity of multiple repeated sessions with the actual available lighting and photosensitizers [57,58,59,60,61,62,63].

#### 4.2.1. Response of Cellular Infiltrate

According to previous research, PDT seems to have the potential to cause a temporary inflammatory response that is predominantly connected with the activation of the immune system [64].

The observation of how PDT not only causes the diversification of new fibroblasts (effector cells) [65], but also promotes the cellular interactions that these cell types have with fibroblast growth factor (FGF)-positive (as well as TNF-alpha) MC cells in their granules, confirms the previous statement.

Therefore, the concept that MCs may send signals that trigger the recruitment and differentiation of new fibroblasts following therapy appears to be plausible [65]. Further supporting this hypothesis is the increase in the degranulation index and the number of these cell types following such therapy. The increased MCs may be attributed to the differentiation of existing precursors inside the tissue, or the influx of precursors that subsequently differentiate into these cells. The subpapillary plexus seems to be a favored location for MC aggregation and cell infiltration during therapy [65]. The activation of the immune system is further supported by the significant expression of TNF-alpha and TGF-beta by mast cells after PDT treatment. TNF-alpha is very important for the development of certain types of dendritic cells, like plasmacytoid cells that interact with regulatory T-type lymphocytes. Following PDT treatment, MCs also express TGF-beta, which is de facto substantial in the differentiation of macrophages. Since this is the case, the reduction in lesion volume that occurs after treatment is certainly connected to the activation of TGF-beta [66]. In truth, it would appear that TGF-beta does have an effect on the epithelial-mesenchymal transition that occurs during the various stages of ulcer healing. This transition is what makes it possible for keratinocytes to move from the borders to the wound bed. In addition, this cytokine has the ability to induce the differentiation of myofibroblasts, which is an important step in the process of scar remodeling [67].

PDT has also been shown to have a significant impact on the activation of neutrophils, which would be a factor in the rise in pro-inflammatory cytokines that would occur after therapy, according to the findings of other studies. Over the course of the acute phase of inflammation resolution and the subsequent restoration of tissue homeostasis, the production of lipid mediators occurs concurrently. Anti-inflammatory and immunomodulatory features are associated with these mediators. Some examples of these properties are the suppression of leukocyte chemotaxis, the blocking of TNF-alpha and IL-6 production, and the subsequent rise in IL-10 expression [60,62,68].

PDT is expected to have both immunostimulatory and immunosuppressive effects, which are likely to be the decisive factor in the type of cell death that is produced. As a result, it is safe to infer that PDT has a significant impact on the immune system.

#### 4.2.2. Neuroimmunomodulation

The capabilities of the nervous system to control the functioning of the immune system are described in [69]. A similar close association can also be seen in the process of ulcer healing. It has been demonstrated through experiments that neurogenic stimuli have a substantial influence on the process of wound repair after an injury has occurred. Furthermore, it has been discovered that delayed wound healing occurs in animal models after the surgical excision of cutaneous nerves [70,71].

Recent studies have shown that the density of neuronal populations in the dermis, which are a component of the autonomic nervous system and contain the typical nerve mediators implicated in ulcer healing (CGRP, NGF, NKA, NPY, SP, PGP 9.5, and VIP), increase after PDT therapy [70,71]. These neuronal populations are found in the dermis. Furthermore, after a single irradiation, there is an increase in the percentage of mast cells that are capable of secreting and containing NGF and VIP compounds. These findings appear to be in agreement with the previously observed rise in the mast cell degranulation index that occurred after PDT therapy. This finding is supported by the fact that VIP and NGF both stimulate mast cell degranulation. Based on this evidence, it appears that this phenomenon might be connected to neurogenic stimuli. Therefore, it is plausible to deduce that mast cell activity after therapy is characterized by an increased release of NGF and VIP, which are capable of stimulating neurons and nerve fibers in the dermis [60,72,73]. This notion is supported by the fact that the aforementioned information has been presented. Conversely, the activation of nerve fibers may be associated with other phenomena, including an increase in the secretion of extracellular matrix (ECM) as well as of TGF-beta, and the response of cellular infiltrate [60,65,66].

Recently, the list of mediators that are involved in the process of wound healing has been expanded to include NO, which is an extracellular molecular messenger. Due to the fact that it is gaseous and has a very short half-life, it is considered to be the smallest known signaling molecule that is capable of freely traversing membranes [74]. The NOS enzyme complex appears to be responsible for the production of this molecule, which is characterized by an overregulation of the inducible isoform in response to stress. In fact, the production of the enzyme is heightened when bacterial antigens, apoptotic bodies, or inflammatory mediators are present. As a consequence of this, it has been hypothesized that iNOS plays a role in the inflammatory phase of wound repair, which is the period in which it increases antibacterial activity and vasodilation [60,75,76]. According to the findings of the research, the expression of iNOS appears to be increased in chronic lesions that are treated with PDT. There is an increase in the degranulation index of mast cells, and these cells also contain iNOS. On the other hand, following therapy, the proportion of these cells that already possess this mediator decreases. On the other hand, the administration of PDT leads to an increase in the expression of iNOS in granulocytes through the treatment. In addition, the amounts of iNOS that are expressed by M1 and M2 macrophages are identical, although the presence of iNOS in blood vessels and fibroblasts is decreased [77].

## 5. Basal Cell Carcinoma

### 5.1. Molecular Biomarkers

Basal cell carcinoma (BCC) is the most common form of skin cancer, arising from the basal cells of the epidermis. It is generally slow-growing and rarely metastasizes; yet it is highly invasive locally, leading to tissue destruction. The development and progression of BCC are influenced by several molecular biomarkers, which are key to understanding its pathogenesis and therapeutic responses [1,78,79,80].

The most well-established molecular pathway in BCC is the Hedgehog (HH) signaling pathway, which plays a pivotal role in the pathogenesis of the disease. Mutations in components of this pathway, particularly Patched1 (PTCH1) and Smoothened (SMO), lead to uncontrolled cell proliferation and tumor formation. PTCH1 is a receptor that inhibits SMO under normal conditions. However, mutations in PTCH1 result in a loss of its inhibitory function, leading to the continuous activation of downstream signaling, which promotes cell cycle progression and survival. Consequently, most BCC cases are driven by these mutations, making the Hedgehog pathway a central biomarker in BCC development [81,82,83].

Another important marker is Cyclin D1, a cell cycle regulator that is upregulated in BCC due to Hedgehog pathway activation. Cyclin D1 promotes cell cycle progression from the G1 to the S phase, facilitating tumor growth. Studies have shown that increased levels of Cyclin D1 are associated with more aggressive BCC subtypes, such as nodular BCC [84].

The Bcl-2 family of proteins, particularly Bcl-2 itself, is implicated in the resistance of BCC cells to apoptosis. Bcl-2 functions as an anti-apoptotic protein, preventing programmed cell death and allowing cancer cells to survive longer, contributing to tumor persistence. Overexpression of Bcl-2 has been observed in both superficial and nodular BCC subtypes, making it a significant molecular marker in the pathology of the disease [1].

Another class of molecules implicated in BCC progression is matrix metalloproteinases (MMPs), particularly MMP-9 and MMP-13. These enzymes degrade extracellular matrix components, facilitating the local invasion of BCC into surrounding tissues. Increased expression of MMPs correlates with the invasive potential of BCC, particularly in more aggressive forms like infiltrative or morphoeic BCC [84].

The tumor suppressor protein p53 also plays a critical role in BCC. While mutations in p53 are not as frequent in BCC as in other skin cancers like squamous cell carcinoma, they do occur and contribute to DNA repair failure and uncontrolled cell proliferation. UV radiation-induced damage to p53 is a common early event in skin carcinogenesis.

### 5.2. Biomolecular Impact of PDT

The efficacy of PDT in BCC treatment is closely linked to its effects on the molecular biomarkers discussed above. Although PDT does not directly target the Hedgehog signaling pathway, its therapeutic effects result in the destruction of cells that are dependent on this pathway for proliferation. Studies show that BCC lesions treated with PDT exhibit decreased levels of Hedgehog pathway activity. PDT-mediated cytotoxicity reduces the population of cells driven by PTCH1 and SMO mutations, leading to tumor shrinkage and resolution in many cases. However, deeper and more aggressive tumors with robust Hedgehog signaling may require adjunctive therapies that directly target this pathway or remove the lesion [43,85,86].

PDT has been shown to reduce Cyclin D1 levels in BCC cells. By generating oxidative stress, PDT disrupts cellular function, leading to apoptosis or necrosis, thereby inhibiting cell cycle progression. As Cyclin D1 is crucial for cell cycle advancement from G1 to S phase, its downregulation following PDT contributes to the cessation of tumor growth [43,87].

One of the primary mechanisms of action of PDT is the induction of apoptosis through the generation of ROS. This process overwhelms the cell’s antioxidant defenses, leading to mitochondrial damage and the activation of apoptotic pathways. Studies have shown that PDT can reduce the expression of anti-apoptotic proteins like Bcl-2, tipping the balance in favor of cell death. The decrease in Bcl-2 expression enhances the sensitivity of BCC cells to apoptosis, making PDT an effective option for inducing programmed cell death in these tumors [43].

PDT has also been shown to affect matrix metalloproteinases, which play a key role in tissue remodeling, inflammation, and tumor progression in BCC. PDT induces the production of reactive oxygen species (ROS), which can activate MMPs, particularly MMP-1, MMP-2, and MMP-9. These enzymes contribute to the breakdown of the extracellular matrix (ECM), facilitating the clearance of tumor cells. However, excessive MMP activation might also lead to tissue damage and inflammation, which can affect the cosmetic outcomes of PDT. Additionally, PDT-induced MMP expression contributes to the healing process post-treatment by aiding in the degradation of damaged tissue and promoting the reformation of new ECM. This dual role of MMPs highlights their importance in both the therapeutic and adverse effects of PDT [84].

## 6. Limitations

This review has several limitations that should be acknowledged. Firstly, the variability of PDT protocols used across different studies, including differences in photosensitizers, light sources, and treatment parameters, makes it challenging to draw consistent conclusions regarding the optimal PDT regimen for different dermatological conditions. Moreover, the lack of standardized biomarker measurement techniques across studies introduces further challenges in comparing results and establishing definitive relationships between biomarkers and treatment outcomes. Lastly, the influence of patient-specific factors such as age, skin type, and comorbidities on PDT efficacy and biomarker response has not been comprehensively addressed, highlighting the need for personalized approaches in future research.

## 7. Conclusions

Photodynamic therapy (PDT) represents a versatile and effective treatment modality for various dermatological conditions, particularly actinic keratosis (AK), non-melanoma skin cancers, skin rejuvenation, and wound healing. The biomolecular and histopathological changes induced by PDT play a critical role in its therapeutic efficacy. Key molecular markers, such as p53, Cyclin D1, Ki-67, Fas/FasL, and survivin, have been studied in the context of AK and non-melanoma skin cancer. These markers not only help in understanding the mechanisms of PDT but also provide insights into its limitations, such as resistance in some cases due to factors like survivin overexpression. By modulating these biomarkers, PDT promotes apoptosis, reduces cellular proliferation, and leads to lesion regression.

PDT induces both apoptotic and necrotic changes in targeted tissues, facilitating the removal of dysplastic keratinocytes in AK and other lesions. It also contributes to the modulation of the extracellular matrix (ECM), enhancing collagen production and tissue remodeling in skin rejuvenation. This effect is mediated through the upregulation of matrix metalloproteinases (MMPs), transforming growth factor-beta (TGF-beta), and other cytokines, which collectively aid in skin regeneration and the restoration of normal skin architecture. While PDT promotes significant reductions in molecular markers like p53 and Ki-67, its impact on markers such as Cyclin D1 may require multiple treatments or combination therapies.

In wound healing, PDT enhances fibroblast activity and cytokine release, leading to better matrix remodeling and improved scar formation. The potential of PDT to modulate immune responses through the Fas/FasL pathway and neuroimmunomodulation also underscores its role in promoting both local and systemic therapeutic effects. However, challenges such as treatment resistance, particularly in more advanced or deeper lesions, suggest the need for further refinement in PDT protocols, including potential combination therapies with agents targeting resistant pathways like survivin.

In conclusion, molecular biomarkers play a crucial role in predicting and enhancing treatment efficacy. By focusing on the former biomarkers, PDT can be tailored to individual patient profiles, paving the way for more personalized and effective treatments. Moreover, the insights gained from this review suggest that advancements in biomarker diagnostics could improve patient selection and enable real-time monitoring of treatment responses, ultimately enhancing therapeutic outcomes. Specific recommendations that can be derived from this review include combining PDT with survivin inhibitors, such as 17-AAG, for lesions with high survivin expression. When considering skin rejuvenation, dermatologists should consider using lower PDT doses for skin to promote fibroblast activation and collagen synthesis while avoiding excessive tissue damage. In cases of chronic wounds, repeated PDT sessions may be necessary to induce sufficient ECM remodeling and enhance healing.

These considerations highlight the importance of tailoring PDT protocols to the specific molecular characteristics of the lesion being treated. Personalized treatment approaches that incorporate biomarker assessments can optimize therapeutic outcomes, improve safety, and potentially reduce treatment resistance.

The integration of molecular diagnostics with PDT has the potential to establish new standards for non-invasive, precise, and effective dermatological therapies, thereby advancing the field of personalized medicine in dermatology.

## Figures and Tables

**Table 1:** Summary of the main biomarkers involved in actinic keratosis and the effect of photodynamic therapy. AK; actinic keratosis, SCC; squamous cell carcinoma, PDT; photodynamic therapy.

Biomarker	Role in AK and SCC	Effect of PDT
p53	Tumor suppressor gene, regulates cell cycle, DNA repair, and apoptosis. Mutated in AK and SCC.	Reduces accumulation of mutated p53. Multiple sessions may be required for complete resolution.
Cyclin D1	Regulates cell cycle transition from G1 to S phase, promoting proliferation. Overexpressed in AK and SCC.	Decreases expression. Multiple treatments may be necessary for full remission.
Ki-67	Marker of cellular proliferation. Higher levels suggest higher aggressiveness.	Significantly reduces Ki-67 expression, reflecting decreased cellular proliferation.
Fas/FasL	Regulates apoptosis through extrinsic apoptotic pathway. Mutations can impair apoptosis.	Upregulates Fas and FasL, activating caspase pathways to promote apoptosis.
Survivin	Inhibits apoptosis by blocking caspase-9 and promotes cell survival. Overexpressed in AK and SCC.	Upregulated after PDT, potentially limiting therapy effectiveness.

**Table 2:** Summary of histological changes and skin biomarkers of PDT regarding skin remodeling.

	Histological Changes	Immunohistochemical Expression
After PDT (0 day–3 weeks)	Monocytic inflammation	Increase in MMP Increase in TGF-beta
Remodeling (3 weeks–3 months)	Lymphocytic infiltrate Dermis fibrosis Elastosis decrease	Increase in MMP-9 Increase in collagen type I
Final stage of remodeling (3–9 months)	Collagen order Elastosis decrease Thinner dermis Flattened epidermis	Decrease in MMP expression Increase of collagen type I and elastin

**Table 3:** Phases, cellular types, molecules, and biomarkers involved in wound healing.

Time	Phases	Cellular Types	Cell Adhesion Molecules	ECM Components	Biomarkers
0–15 min	Hemostasis	Endothelial cells, platelets		Fibrin, fibronectin	Epinephrine, prostaglandins, thromboxanes, thrombin
15 min–6 days	Inflammation	Endothelial cells, mast cells, dendritic cells, macrophages, T lymphocytes		Temporary matrix formed by fibrin, complement proteins, PDGF, IL-8, IL-1 alpha, IL-1 beta, IL-6 and TNF-alpha	Sympathetic nervous system, histamine, kinins, leukotrienes, thrombin
24–48 h–7 days	Proliferation	Endothelial cells, fibroblasts, keratinocytes,	Alphabeta-3, Beta-1-integrins, integrins	Collagen, fibronectin, GAGs, proteoglycans, Tenascin, Vitronectin (temporary ECM)	Angiopoietin, MMPs/TIMP, FGF-2, FGF-7, FGF-10, GM-CSF, NO, TGF-beta, NGF, HGF, HB-EGF, IL-6, Leptin, PDGF, VEGF
From 2 days to several weeks	Maturation	Fibroblasts, myofibroblasts	Integrins	Collagen	EGF, IGF, FGF-2, NGF, PDGF, TGF-beta

## Data Availability

The original contributions presented in the study are included in the article, further inquiries can be directed to the corresponding author.
